# Clinical and epidemiological characteristics of patients in the head and neck surgery department of a university hospital

**DOI:** 10.1590/S1516-31802012000500007

**Published:** 2012-11-13

**Authors:** Maurício José Cabral Ruback, Ana Lívia Galbiatti, Lidia Maria Rebolho Batista Arantes, Gustavo Henrique Marucci, Anelise Russo, Mariangela Torreglosa Ruiz-Cintra, Luiz Sérgio Raposo, José Victor Maniglia, Érika Cristina Pavarino, Eny Maria Goloni-Bertollo

**Affiliations:** I MD, MSc. Master’s Student. Unidade de Pesquisa em Genética e Biologia Molecular (UPGEM), Faculdade de Medicina de São José do Rio Preto (Famerp), São José do Rio Preto, São Paulo, Brazil.; II PhD. Adjunct Professor, Department of Biological Sciences, Universidade Federal do Triângulo Mineiro (UFTM), Uberaba, Minas Gerais, Brazil.; III MD, MSc. Professor, Department of Otolaryngology and Head and Neck Surgery, Faculdade de Medicina de São José do Rio Preto (Famerp), São José do Rio Preto, São Paulo, Brazil.; IV MD, PhD. Adjunct Professor, Department of Otolaryngology and Head and Neck Surgery, Faculdade de Medicina de São José do Rio Preto (Famerp), São José do Rio Preto, São Paulo, Brazil.; V PhD. Adjunt Professor, Department of Molecular Biology, Faculdade de Medicina de São José do Rio Preto (Famerp), São José do Rio Preto, São Paulo, Brazil

**Keywords:** Epidemiology, Head and neck neoplasms, Habits, Alcoholism, Tobacco, Epidemiologia, Neoplasias de cabeça e pescoço, Hábitos, Alcoolismo, Tabaco

## Abstract

**CONTEXT AND OBJECTIVES:**

Head and neck cancer is the fifth most common type of cancer worldwide. The objective of this study was to evaluate the clinical and epidemiological parameters in a head and neck surgery service.

**DESIGN AND SETTING:**

Cross-sectional study using patients’ records, developed in otolaryngology and head and neck department of a university hospital in the northwest of the state of São Paulo.

**METHODS:**

A total of 995 patients in the head and neck surgery service between January 2000 and May 2010 were evaluated. The variables analyzed included: age, gender, skin color, tobacco and alcohol consumption, primary site, staging and histological tumor type, treatment and number of deaths.

**RESULTS:**

The disease was more frequent among men (79.70%), smokers (75.15%) and alcohol abusers (58.25%). The most representative sites were oral cavity (29.65%) and larynx (24.12%) for the primary site; squamous cell carcinoma (84.92%) was the most frequent histological type, and surgery (29.04%) and radiotherapy (14.19%) were the most common treatments.

**CONCLUSION:**

The cancer that affects patients assisted by the head and neck surgery service occurs mainly men, smokers and alcohol abusers, and the oral cavity and larynx are the sites with the highest incidence. The high rate of patients with stages III and IV indicates late diagnosis by the treatment centers, which reflects the need for prevention education campaigns for early diagnosis of the disease.

## RESUMO

**CONTEXTO E OBJETIVOS:**

O câncer de cabeça e pescoço é o quinto tipo mais comum entre todas as neoplasias no mundo. O objetivo do estudo foi avaliar os parâmetros clínicos e epidemiológicos em um serviço de cirurgia de cabeça e pescoço.

**TIPO DE ESTUDO E LOCAL:**

Estudo transversal com coleta de dados de prontuários, realizado no departamento de otorrinolaringologia e cabeça e pescoço de um hospital universitário do noroeste do estado de São Paulo.

**MÉTODOS:**

Um total de 995 pacientes do serviço de cirurgia de cabeça e pescoço foi avaliado entre janeiro de 2000 a maio de 2010. As variáveis analisadas foram: idade, gênero, cor da pele, consumo de álcool e tabaco, sítio primário, estádio e tipo histológico do tumor, tratamento e número de mortes.

**RESULTADOS:**

A doença foi mais frequente entre homens (79,70%), tabagistas (75,15%) e etilistas (58,25%). Os locais mais representativos foram: cavidade oral (29,65%) e laringe (24,12%) para sítio primário; carcinoma espinocelular (84,92%) foi o tipo histológico mais frequente e cirurgia (29,04%) e radioterapia (14,19%) foram os tratamentos mais comuns.

**CONCLUSÃO:**

O câncer que afeta os pacientes assistidos pelo serviço de cirurgia de cabeça e pescoço ocorre, em sua maioria, entre homens, tabagistas e etilistas, tendo a cavidade oral e a laringe maior incidência. A alta taxa de pacientes com estádios III e IV indica diagnóstico tardio pelos centros de tratamento, o que reflete a necessidade de campanhas de prevenção para o diagnóstico precoce da doença.

## INTRODUCTION

Head and neck cancer involves a broad category of diverse tumor types arising from various anatomical structures including the craniofacial bones, soft tissues, salivary glands, skin and mucosal membranes.^1^

Although head and neck surgery departments treat patients with malignant tumors of the upper aerodigestive tract, skin and thyroid, the term “head and neck cancer” is frequently used for the group of neoplasms located in the upper aerodigestive tract: approximately 40% of them occur in the oral cavity, 15% in the pharynx, 25% in larynx and 20% at other anatomical sites.^2^^,^^3^ Approximately 95% of these tumors have squamous cell carcinoma as the primary histological type.^4^

Head and neck cancer is the fifth most common type of cancer worldwide, among all neoplasms.^4^ The overall survival rate for this cancer is variable, depending on the primary site and disease stage. For oral cavity cancer, the overall survival rate is 50% over five years.^5^ For other sites (pharynx and larynx), the rate is greater than 50% for early stage disease (T1-T2, N0) and generally less than 50% at advanced stages (T3-T4, N0, T3-T4, N+, or any T and N2-N3).^6^ In 2009, 6,530 deaths were registered in Brazil as a result of oral cancer, being 5,136 for men and 1,394 for women.^7^

Oral cavity cancer is the most representative type of the disease, and has a high frequency in Southeast Asia and India, due to the habit within these regions of chewing tobacco leaf and betel nut. The latter is a stimulant commonly used among Indians.^8^ In the United States, about 21,000 new cases of oral cancer are diagnosed each year and it has been estimated that more than 650,000 new cases of head and neck cancer are diagnosed each year worldwide, two-thirds of them in developed countries.^6^

The estimated numbers of new cases of oral cavity cancer for 2012 were 14,170 and 4,430 for Brazil and the state of São Paulo, respectively.^7^ Preliminary research conducted by our group in a reference hospital in the northwestern part of the state of São Paulo over a five-year period showed 427 patients diagnosed with head and neck cancer.^9^

This tumor type occurs mainly in male individuals, and its occurrence increases with age.^10^ Over the last decade, there has been a significant increase in this cancer among younger individuals, possibly due to increased numbers of infections by the human papillomavirus (HPV).^6^^,^^11^^,^^12^

Head and neck cancer develops as the result of interactions between environmental factors and genetic inheritance, and is therefore multifactorial. Tobacco use associated with alcohol consumption is a well-established risk factor for head and neck cancer.^13^ Alcohol can act as a solvent for some tobacco carcinogens, thus increasing cellular uptake of these substances. According to Marur and Forastiere,^6^ tobacco consumption associated with alcohol consumption increases the head and neck cancer risk 40-fold.

A significant proportion of head and neck tumors result from infection by some HPV types. These viruses affect cells and produce viral oncoproteins (E6 and E7) that promote tumor progression by inactivating tumor suppressor genes such as Tp53 (tumor protein 53) and pRb (retinoblastoma tumor suppressor gene).^12^ Other factors that may contribute towards head and neck carcinogenesis include diet, in which the risk is reduced through fruit and vegetable consumption;^14^ oral hygiene, in which the risk is higher with inadequate care, thereby leading to chronic infections caused by the bacteria responsible for the pathogenesis of this tumor type;^15^ and body mass, which can modulate toxin and carcinogen metabolism.^16^

Occupational activity also appears to be associated with head and neck cancer development. The study by Conway et al.^17^ showed that manual occupational activities, low income, low occupational-social class, low educational attainment and unemployment correlate with increased risk of disease development. Individuals who work in rural activities are constantly exposed to sunlight and in contact with carcinogenic substances that contribute towards the development of oral cavity cancer.^18^

Skin cancer is also associated with excessive exposure to solar radiation and occurs more frequently in the portions of the body exposed to the sun (head, neck and limbs). Also influencing the appearance of these lesions are factors such as age, sex, ethnicity, smoking, alcohol abuse, geographical distribution, old scars, persistent physical aggression and exposure to radioactive substances.^19^

Thyroid gland tumors, with their own unique characteristics, but also treated in head and neck surgery departments, have an origin relating to iodine deficiency, external beam radiotherapy during childhood and adolescence, exposure to ionizing radiation and preexisting thyroid disease.^20^

## OBJECTIVE

The purpose of this study was to describe the sociodemographic and clinical-pathological characteristics of patients treated in the head and neck surgery department of a university hospital in the northwestern part of the state of São Paulo, between January 2000 and May 2010.

## METHODS

We conducted a cross-sectional study using patients’ records. The medical records of 1,351 cancer patients treated at the Otolaryngology and Head and Neck department of a university hospital in the northwest of the state of São Paulo, which has 16 resident physicians, 20 medical students, 4 teachers and 10 nurses, between January 2000 and May 2010, were evaluated. The study protocol was approved by the National Ethics Committee (CONEP-5566/2005; SISNEP 0976.0.140.000-05).

The variables analyzed were age, gender, skin color, tobacco and alcohol consumption, primary site, histological type, staging, treatment, death and occupational therapy, among patients with upper aerodigestive tract, skin and thyroid cancer. Individuals who had smoked more than 100 cigarettes in their lifetimes were considered to be smokers, and individuals who were consuming at least four drinks per week were considered to be alcohol drinkers.^21^^,^^22^

Tumors of the upper aerodigestive tract were classified according to the anatomical site in the oral cavity, pharynx, larynx, nasal cavity or salivary glands, or as unknown primary site. Skin tumors and thyroid cases were also included.

Clinical staging of patients was performed according to the criteria of the International Union Against Cancer, based on its classification of malignant tumors (TNM).^23^ In these standards for classification stages, T represents the tumor size, and tumors classified as Tx and T0 indicate primary indefinite tumor and no signs of primary tumor, respectively. N1, N2 and N3 indicate presence of regional lymph nodes involvement and N0 indicates their absence. Tumors classified as Nx indicate undetermined lymph node status. Metastases are represented by M1 and M0 for absence. In cases of failure to diagnose the presence or absence of metastasis, tumors are classified as Mx.

The patients’ occupations were classified into sectors such as agriculture, construction, domestic service, driving, commerce, administration, surveillance, metalwork, tapestries and aesthetics.

The data were analyzed by descriptive statistics, using the Excel software (version 2007).

## RESULTS

We analyzed 1,351 patients’ records with comprehensive information. Complete data was obtained for 995 cases and in relation to tumor staging and treatment 785 and 909 records were complete, respectively.

A substantial majority of the cases were male (79.68%) and the mean age of patients was 60.48 years. Out of the total number of patients, 747 were smokers (75.15%), 579 were alcohol abusers (58.25%) and 547 (54.00%) were smokers and alcohol abusers. For skin color classification, the subjects were divided into white and nonwhite, according to their medical records, and it was found that 90.04% of the patients had white skin. The oral cavity was the most representative primary site of occurrence (29.65%), followed by the larynx and pharynx (24.12% and 18.29%, respectively). Thyroid gland tumors accounted for 5.43% of the cases, while skin tumors accounted for 6.83%. In the cases of 110 patients, it was not possible to identify the primary site (Table 1). The predominant histological type was squamous cell carcinoma, representing 84.92% of the cases, followed by basal cell carcinoma (6.03%) and papillary carcinoma (5.22%). Other types of malignant tumors such as adenocarcinoma, melanoma, sarcoma, chondrosarcoma, fibrosarcoma and follicular carcinoma accounted for 3.08% (Table 2).

**Table 1.** Distribution of patients according to demographic characteristics and tumor sites

Variables	Number of patients (%)
Gender	
Male	793 (79.70)
Female	202 (20.30)
Skin color	
White	895 (90.04)
Nonwhite	99 (9.96)
Tobacco use	
Yes	747 (75.15)
No	247 (24.85)
Alcohol use	
Yes	579 (58.25)
No	415 (41.75)
Tobacco and alcohol use	547 (54.00)
Tumor sites	
Oral cavity	295 (29.65)
Larynx	240 (24.12)
Pharynx	182 (18.29)
Skin	68 (6.83)
Thyroid	54 (5.43)
Nasal cavity	16 (1.61)
Other sites	30 (3.01)
Unknown primary site	110 (11.06)

**Table 2.** Most frequent histological types among patients attending a head and neck surgery department

Histological types	Number of patients (%)
Squamous cell carcinoma	845 (84.92)
Basal cell carcinoma	60 (6.03)
Papillary carcinoma	52 (5.22)
Other	38 (3.83)

The tumor stage (TNM) in relation to primary sites and the main methods of treatment received by patients are described in Tables 3 and 4, respectively. Regarding the patients’ occupations, the main activity related to agriculture, and was represented by 207 patients (20.82%).

**Table 3.** Distribution of the clinical histopathological parameters of cancer patients treated at an otolaryngology and head and neck department

Category	Tumor staging					
	T1 and T2 n (%)	T3 and T4 n (%)	N0 n (%)	N1, N2 and N3 n (%)	M0 n (%)	M1 n (%)
Oral cavity	169 (40.14)	102 (31.19)	202 (37.75)	69 (25.46)	249 (33.73)	2 (28.57)
Larynx	105 (24.94)	114 (34.86)	160 (29.90)	64 (23.62)	208 (28.18)	-----
Pharynx	66 (15.68)	98 (27.97)	89 (16.60)	78 (28.78)	141 (19.10)	2 (28.57)
Skin	39 (9.26)	1 (0.31)	41 (7.66)	-----	41 (5.55)	-----
Thyroid	24 (5.70)	7 (2.14)	23 (4.29)	5 (1.85)	28 (3.79)	-----
Nasal cavity	5 (1.19)	3 (0.92)	7 (1.13)	1 (0.37)	8 (1.08)	-----
Other sites	13 (3.09)	2 (0.61)	13 (2.49)	3 (1.11)	15 (2.03)	-----
Unknown site	0 (0)	0 (0)	0 (0)	51 (18.82)	48 (6.54)	3 (42.86)

**Table 4.** Forms of treatment administered to cancer patients treated at an otolaryngology and head and neck department

Treatment	Number of patients (%)
Surgery	264 (29.04)
Radiotherapy	129 (14.19)
Chemotherapy	18 (1.98)
Iodotherapy	1 (0.11)
Surgery and iodotherapy	23 (2.53)
Surgery and radiotherapy	272 (29.92)
Surgery and chemotherapy	17 (1.87)
Radiotherapy and chemotherapy	59 (6.49)
Surgery, radiotherapy and chemotherapy	126 (13.86)

## DISCUSSION

In the present study, the patients’ mean age, regardless of gender, was 60.48 years, i.e. similar to what was observed in other Brazilian^24^ and American^25^ populations. Although the frequency of patients with head and neck cancer is higher among individuals with advanced age, increasing numbers of cases among young people have been observed, especially for oral cavity and oropharynx cancer and in association with HPV 16 infection.^12^ The latter is etiologically age-related to the carcinogenesis of this tumor type.^11^

Head and neck cancer predominantly affects male.^9^^,^^26^^,^^27^^,^^28^ This was corroborated in the present study, in which 79.68% of the individuals affected by this type of cancer were male. Despite the low incidence of malignancies among women, increasing numbers of cases are expected as a result of increasing tobacco and alcohol consumption among the female population.^29^

In the present study, a high prevalence of white skin individuals (90.04%) was observed, similar to the findings from a study conducted recently in the southern states of Brazil^30^ and the Midwest of the United States.^31^ However, Hayat et al.^32^ observed a higher prevalence of head and neck cancer among African-American descendants from different areas of the United States. The ethnic differences in distribution found among patients with head and neck cancer in different studies are mainly due to the population composition where the research was done. Our study was conducted in the northwestern part of the state of São Paulo, which was predominantly colonized by Europeans, such that a high percentage of the population is white-skinned.

The tobacco consumption rate was 75.15%, the alcohol consumption rate was 58.25% and the rate for both together was 54.00%, among the patients studied. It has been well established that alcohol and tobacco consumption is associated with head and neck cancer, and this has been reported in several studies.^13^^,^^26^^,^^33^ An association between experiencing passive smoking for over 15 years and development of head and neck cancer has been reported, independent of alcohol consumption.^34^

Squamous cell carcinoma was the most common histological type, representing 84.82% of the cases, with a frequency close to what is observed in the literature, which is approximately 90%.^5^ In relation to primary tumor site, the oral cavity was the most representative site (29.65%), followed by the larynx (24.12%) and pharynx (18.29%). The incidence of oral cancer worldwide is higher than at other anatomical sites, and this type of cancer is more common in individuals with low income, low occupational social class and low educational attainment.^35^ Although it was not possible to obtain full information in our study, most of the patients treated in the hospital came from the public health system, which serves low-income patients.

Surgical procedures and exclusive radiotherapy were performed on 29.04% and 14.19% of the patients, respectively. Surgery followed by radiotherapy is a common practice in treating head and neck squamous cell carcinoma, especially in the early stages of the disease (I or II), with a high percentage of cure.^36^ In this study, both treatment procedures were used in 29.92% of the cases. At advanced stages (III or IV), chemotherapy is usually used in conjunction with other forms of treatment, thus promoting, especially in conjunction with radiotherapy, increased locoregional control in many cases.^36^ Among the patients analyzed in this study, 13.86% underwent surgical, radiotherapy and chemotherapy procedures.

Patients with skin tumors in our study were treated surgically, which provides cure rates greater than 95% when the treatment is administered early and properly.^20^ Regarding thyroid gland tumors, our results indicated that surgery was the most prevalent treatment, sometimes associated with iodine therapy as an optional treatment. Use of less conservative surgery and lymph node exploration, as well as the use of adjuvant iodine therapy, seem to determine a more favorable prognosis for patients with thyroid cancer.^37^

In analyzing the tumor stage, a high proportion of tumors classified as T3 and T4 was observed. These data reveal that a significant number of patients were only diagnosed at advanced stages of the disease and demonstrate the difficulty in obtaining an early diagnosis, since symptoms rarely appear in the early stages. According to the literature, on average, 40% of patients with oral cancer are diagnosed in advanced stages.^38^ In Brazil, some studies have reported that less than 50% of the patients receive an early diagnosis.^39^^,^^40^ Nearly two-thirds of patients with head and neck cancer have an advanced stage of the disease, usually involving regional lymph nodes. The incidence of distant metastases is relatively small in cases of malignant tumors of head and neck cancer, compared with other regions.^41^ Approximately one-third of the patients in this study had lymph node involvement, while only 1% presented metastases.

During the study period, 265 patients died. The high mortality rate for this tumor type has remained virtually constant over the last few decades.^11^ Nevertheless, Zigon et al.^27^ observed an increase in the relative rate of five-year survival among cases of head and neck cancer, in a study on a European population. In another study on a Brazilian population, we observed a significant increase in the five-year survival rate for oral and oropharyngeal cancer, ranging from 28.7% among patients treated in the 1950s to 43.2% in the 1990s.^40^

Regarding the occupational activities performed by our study subjects, the most frequent ones related to agriculture (20.82%) and construction (19.01%). This is consistent with the findings of Conway et al.,^35^ who showed a correlation between higher rates of head and neck cancer and individuals who do manual occupational activities and are in a low occupational social class.

Sartor et al.^42^ showed that there was an association between laryngeal cancer and exposure to respirable free crystalline silica. Wünsch Filho^43^ also showed that low educational levels and some occupations are associated with a high risk of laryngeal cancer. A large proportion of the patients with laryngeal cancer are workers exposed to a variety of chemical hazards like polycyclic aromatic compounds, cement dust, metal dust, asbestos, varnish and lacquer. In this study, we found that the risk was twice as high for exposed individuals, such as those working in construction, compared with unexposed individuals.

Recent research within molecular biology has improved our understanding of the etiology of these tumors. Combination of prognostic factors with molecular parameters could be beneficial for patients, through application of new therapeutic strategies. Such advances in studies on molecular markers may also improve the diagnosis at early stages of the disease, which would not be possible with traditional clinical methods.^26^^,^^44^ It is not known to what extent established prognostic factors such as late stage presented at diagnosis, the presence and severity of comorbidities or differences in cancer treatment account for the excessive mortality among patients with head and neck cancer. Therefore, control measures are needed, such as health promotion programs relating to controlling tobacco and alcohol consumption and carcinogen exposure in occupational settings. Moreover, studies on clinical responses may provide benefits for patients and new information for estimating prognoses, making treatment decisions and enabling differentiated services for each patient.

## CONCLUSION

Malignant tumors treated at the head and neck surgery department of a university hospital in the northwest of the state of São Paulo occurred more frequently among subjects from regions with low socioeconomic development, and with limited access to education. The incidence of this disease in this specific population mostly affected male patients in their sixties, smokers and alcohol abusers, and the oral cavity and larynx were the regions most affected. The high rate of patients with stages III and IV indicated late demand in treatment centers, which reflects the need for preventive education campaigns in order to achieve early diagnosis of the disease.

## References

[1] Pai SI, Westra WH (2009). Molecular pathology of head and neck cancer: implications for diagnosis, prognosis, and treatment. Annu Rev Pathol.

[2] Döbrossy L (2005). Epidemiology of head and neck cancer: magnitude of the problem. Cancer Metastasis Rev.

[3] Lee KJ (2003). Essential otolaryngology: head & neck surgery.

[4] Marcu LG, Yeoh E (2009). A review of risk factors and genetic alterations in head and neck carcinogenesis and implications for current and future approaches to treatment. J Cancer Res Clin Oncol.

[5] Walker DM, Boey G, McDonald LA (2003). The pathology of oral cancer. Pathology.

[6] Marur S, Forastiere AA (2008). Head and neck cancer: changing epidemiology, diagnosis, and treatment. Mayo Clin Proc.

[7] Instituto Nacional do Câncer Boca.

[8] Kanavos P (2006). The rising burden of cancer in the developing world. Ann Oncol.

[9] Alvarenga L de M, Ruiz MT, Pavarino-Bertelli EC (2008). Epidemiologic evaluation of head and neck patients in a university hospital of Northwestern São Paulo State. Braz J Otorhinolaryngol.

[10] Crozier E, Sumer BD (2010). Head and neck cancer. Med Clin North Am.

[11] Gillison ML, D'Souza G, Westra W (2008). Distinct risk factor profiles for human papillomavirus type 16-positive and human papillomavirus type 16-negative head and neck cancers. J Natl Cancer Inst.

[12] Hennessey PT, Westra WH, Califano JA (2009). Human papillomavirus and head and neck squamous cell carcinoma recent evidence and clinical implications. J Dent Res.

[13] Boffetta P, Hashibe M (2006). Alcohol and cancer. Lancet Oncol.

[14] Pavia M, Pileggi C, Nobile CG, Angelillo IF (2006). Association between fruit and vegetable consumption and oral cancer: a meta-analysis of observational studies. Am J Clin Nutr.

[15] Guha N, Boffetta P, Wünsch V (2007). Oral health and risk of squamous cell carcinoma of the head and neck and esophagus: results of two multicentric case-control studies. Am J Epidemiol.

[16] Peters ES, Luckett BG, Applebaum KM (2008). Dairy products, leanness, and head and neck squamous cell carcinoma. Head Neck.

[17] Conway DI, McMahon AD, Smith K (2010). Components of socioeconomic risk associated with head and neck cancer: a population-based case-control study in Scotland. Br J Oral Maxillofac Surg.

[18] Santos LC, Cangussu MC, Batista Ode M, Santos JP (2009). Oral cancer population sample of the state of Alagoas at a reference hospital. Braz J Otorhinolaryngol.

[19] Dergham AP, Muraro CC, Ramos EA, Mesquita LAF, Collaço LM (2004). Distribuição dos diagnósticos de lesões pré-neoplásicas e neoplásicas de pele no Hospital Universitário Evangélico de Curitiba [Distribution of diagnosis of neoplastic and preneoplastic skin lesions at Evangelical Hospital in Curitiba]. An Bras Dermatol.

[20] Golbert L, Wajner SM, Rocha AP, Maia AL, Gross JL (2005). Carcinoma diferenciado de tireóide: avaliação inicial e acompanhamento [Differentiated thyroid carcinoma: initial evaluation and follow-up]. Arq Bras Endocrinol Metabol.

[21] Ahrendt SA, Chow JT, Yang SC (2000). Alcohol consumption and cigarette smoking increase the frequency of p53 mutations in non-small cell lung cancer. Cancer Res.

[22] Kjaerheim K, Gaard M, Andersen A (1998). The role of alcohol, tobacco, and dietary factors in upper aerogastric tract cancer: a prospective study of 10,900 Norwegian men. Cancer Causes Control.

[23] Sobin LH, Wittelind CH (2002). TNM classification of malignant tumors.

[24] Vilela LD, Allison PJ (2010). An investigation of the role of sense of coherence in predicting survival among Brazilians with head and neck cancer. Oral Oncol.

[25] Potash AE, Karnell LH, Christensen AJ, Vander Weg MW, Funk GF (2010). Continued alcohol use in patients with head and neck cancer. Head Neck.

[26] Galbiatti AL, Ruiz MT, Biselli-Chicote PM (2010). 5-Methyltetrahydrofolate-homocysteine methyltransferase gene polymorphism (MTR) and risk of head and neck cancer. Braz J Med Biol Res.

[27] Zigon G, Berrino F, Gatta G (2011). Prognoses for head and neck cancers in Europe diagnosed in 1995-1999: a population-based study. Ann Oncol.

[28] St Guily JL, Borget I, Vainchtock A, Rémy V, Takizawa C (2010). Head and neck cancers in France: an analysis of the hospital medical information system (PMSI) database. Head Neck Oncol.

[29] La Vecchia C, Lucchini F, Negri E, Levi F (2004). Trends in oral cancer mortality in Europe. Oral Oncol.

[30] Silver HJ, de Campos Graf Guimaraes C, Pedruzzi P (2010). Predictors of functional decline in locally advanced head and neck cancer patients from south Brazil. Head Neck.

[31] Shuman AG, Entezami P, Chernin AS (2010). Demographics and efficacy of head and neck cancer screening. Otolaryngol Head Neck Surg.

[32] Hayat MJ, Howlader N, Reichman ME, Edwards BK (2007). Cancer statistics, trends, and multiple primary cancer analyses from the Surveillance, Epidemiology, and End Results (SEER) Program. Oncologist.

[33] Hashibe M, Boffetta P, Zaridze D (2007). Contribution of tobacco and alcohol to the high rates of squamous cell carcinoma of the supraglottis and glottis in Central Europe. Am J Epidemiol.

[34] Lee YC, Boffetta P, Sturgis EM (2008). Involuntary smoking and head and neck cancer risk: pooled analysis in the International Head and Neck Cancer Epidemiology Consortium. Cancer Epidemiol Biomarkers Prev.

[35] Conway DI, Petticrew M, Marlborough H (2008). Socioeconomic inequalities and oral cancer risk: a systematic review and meta-analysis of case-control studies. Int J Cancer.

[36] Argiris A, Karamouzis MV, Raben D, Ferris RL (2008). Head and neck cancer. Lancet.

[37] Rouxel A, Hejblum G, Bernier MO (2004). Prognostic factors associated with the survival of patients developing loco-regional recurrences of differentiated thyroid carcinomas. J Clin Endocrinol Metab.

[38] Rogers SN, Pabla R, McSorley A (2007). An assessment of deprivation as a factor in the delays in presentation, diagnosis and treatment in patients with oral and oropharyngeal squamous cell carcinoma. Oral Oncol.

[39] Wünsch-Filho V (2002). The epidemiology of oral and pharynx cancer in Brazil. Oral Oncol.

[40] Carvalho AL, Ikeda MK, Magrin J, Kowalski LP (2004). Trends of oral and oropharyngeal cancer survival over five decades in 3267 patients treated in a single institution. Oral Oncol.

[41] Ferlito A, Shaha AR, Silver CE, Rinaldo A, Mondin V (2001). Incidence and sites of distant metastases from head and neck cancer. ORL J Otorhinolaryngol Relat Spec.

[42] Sartor SG, Eluf-Neto J, Travier N (2007). Riscos ocupacionais para o câncer de laringe: um estudo caso-controle [Occupational risks for laryngeal cancer: a case-control study]. Cad Saude Publica.

[43] Wünsch V (2004). The epidemiology of laryngeal cancer in Brazil. Sao Paulo Med J.

[44] Ruiz MT, Pavarino-Bertelli E, Maniglia JV, Ruback MJC, Goloni-Bertollo EM (2006). Epidemiologia e biomarcadores de em câncer de cabeça e pescoço [Head and neck cancer epidemiology and biomarkers]. Arq Ciênc Saúde.

